# Effects of Petroleum-Based Oils as Dispersing Aids on Physicochemical Characteristics of Magnetorheological Elastomers

**DOI:** 10.3390/ma14227026

**Published:** 2021-11-19

**Authors:** Norizatie Muhammad Zaki, Nurul Azhani Yunus, Muhamad Shakir Yusoff, Saiful Amri Mazlan, Siti Aishah Abdul Aziz, Nor Aziyatul Izni, Irfan Bahiuddin

**Affiliations:** 1 Department of Mechanical Engineering, Universiti Teknologi PETRONAS, Seri Iskandar 32610, Malaysia; zatie_nori@yahoo.com (N.M.Z.); shakiryusoff889@gmail.com (M.S.Y.); 2 Engineering Materials and Structures (eMast) iKohza, Malaysia-Japan International Institute of Technology (MJIIT), Universiti Teknologi Malaysia, Jalan Sultan Yahya Petra, Kuala Lumpur 54100, Malaysia; amri.kl@utm.my (S.A.M.); aishah118@gmail.com (S.A.A.A.); 3 Department of Actuarial Science & Applied Statistics, Faculty of Business and Management, UCSI University Kuala Lumpur (South Wing), Cheras, Kuala Lumpur 56000, Malaysia; aziyatul@ucsiuniversity.edu.my; 4 Department of Mechanical Engineering, Vocational College, Universitas Gadjah Mada, Jl. Yacaranda Sekip Unit IV, Yogyakarta 55281, Indonesia; irfan.bahiuddin@ugm.ac.id

**Keywords:** magnetorheological elastomers, petroleum-based oil, dispersing aids, natural rubber, physicochemical characteristics

## Abstract

This paper investigated the effects of petroleum-based oils (PBOs) as a dispersing aid on the physicochemical characteristics of natural rubber (NR)-based magnetorheological elastomers (MREs). The addition of PBOs was expected to overcome the low performance of magnetorheological (MR) elastomers due to their inhomogeneous dispersion and the mobility of magnetic particles within the elastomer matrix. The NR-based MREs were firstly fabricated by mixing the NR compounds homogeneously with different ratios of naphthenic oil (NO), light mineral oil (LMO), and paraffin oil (PO) to aromatic oil (AO), with weight percentage ratios of 100:0, 70:30, 50:50, and 30:70, respectively. From the obtained results, the ratios of NO mixed with low amounts of AO improved the material physicochemical characteristics, such as thermal properties. Meanwhile, LMO mixed the AO led to the best results for curing characteristics, microstructure observation, and magnetic properties of the MREs. We found that the LMO mixed with a high content of AO could provide good compatibility between the rubber molecular and magnetic particles due to similar chemical structures, which apparently enhance the physicochemical characteristics of MREs. In conclusion, the 30:70 ratio of LMO:AO is considered the preferable dispersing aid for MREs due to structural compounds present in the oil that enhance the physicochemical characteristics of the NR-based MREs.

## 1. Introduction

Magnetorheological elastomers (MREs) are a kind of smart material, whose rheological properties can be continuously, reversely, and rapidly controlled by an external magnetic field due to the existence of magnetic particles embedded in the elastomer matrix [1,2,3,4,5,6,7]. Over the past decade, the properties of MREs have attracted immense attention, and they have use in several applications, such as prosthetic devices, spring elements, automotive bushing, medical devices, vibration absorbers, and piezo-sensors [8,9,10]. MREs can be fabricated as anisotropic or isotropic; in the former, a magnetic field is applied during the curing process; for isotropic, the curing process is performed without a magnetic field, producing either a uniform or random distribution of magnetic particles. Meanwhile, anisotropic MREs contain chain-like or columnar structures of magnetic particles [11,12].

The matrix, magnetic particles, and additives are the main components of MREs, all of which have shown great influences on their properties. Most MRE research groups used NR and synthetic rubbers such as silicone rubber (SiR) and polyurethane rubber (PU) as matrices. According to Jonsdottir et al. [9], PU showed agglomeration in the sample due to difficulty in blending well with magnetic particles. SiR faces a problem when magnetic particles are implemented in the MREs, since the particles cannot easily be blended with an SiR matrix [13,14]. In addition, SiR is soft rubber, and consequently lacks mechanical properties such as low impact loading, which limits its application [15]. For instance, Chen et al. [16] found that NR is better used as a matrix elastomer in terms of mechanical properties than SiR—known as soft rubber. Hence, it was observed that SiR is not suitable for application due to its difficulty in withstanding harsh conditions such as earthquakes. NR was chosen as the alternative matrix in this study, since it is well known that NR exhibits an ideal matrix for excellent MRE performances such as good dynamic performance, high elasticity, and resilience over a wide temperature range [17,18,19]. Carbonyl iron particles (CIPs), known as soft ferromagnetic materials, are often used as magnetic particles due to their desirable magnetic properties, including high magnetic permeability, high magnetic saturation, and low magnetic remanence [18,20]. The particle distribution within MREs has a significant impact on MR effects and other MRE properties—particularly rheological properties—as described by previous research [21,22]. Based on the feasibility studies performed by many MRE research groups, the magnetic particles are inhomogeneously dispersed in the matrix because of high viscosity NR [19,23]. For instance, Jung et al. [17] fabricated the MREs in two different forms of particle distribution—isotropic and anisotropic—using NR as the matrix. They found that the anisotropic MRE has a larger storage modulus than the isotropic.

In addition to the inhomogeneous distribution of particles, the wettability of the particles within the elastomer matrix also affects the MREs’ properties. For example, Cvek et al. [24] found that coating the CIPs with poly(trimethylsilyloxyethyl methacrylate) (PHEMATMS) significantly enhanced the particle mobility in the elastomer matrix. Moreover, the polymer coating improved the thermo-oxidation stability and anti-acid/corrosion properties. In the meantime, the result obtained also exhibited a higher damping factor due to increases in particle mobility and MR effect, which are associated with practical applications. On the other hand, some other studies have shown that the use of a dispersing aid for unsaturated rubber matrices such as NR could enhance the rubber’s flexibility, reduce the viscosity of the matrix, and improve processability [25,26]. Generally, aromatic oil (AO) is the most widely used dispersing aid in rubber compounds due to its good compatibility with the rubber and ability as a softening agent to improve the workability of rubber composition [27]. Similarly, NR-based MREs use AO as a dispersing aid. For example, in the study of Syamin et al. [27], different oils—including cooking oil, AO, and paraffin oil (PO)—were used as processing oils with rubber compounds. The results showed that AO indicated a longer scorch time than cooking oil and PO with NR, providing slightly better processing safety and leading to to better mechanical properties. The physical properties of the vulcanizates for cooking oil were almost similar to those of AO and PO, except for the curing characteristics.

The drawback of AO is that it consists of unsaturated aromatic hydrocarbons and has a double bond, which is more reactive. Hence, the crosslink density decreases, adversely affecting the physical and mechanical properties of the rubber. Based on these properties, AO may have the potential to improve the MR performance of MREs when mixed with other PBOs [10,25,28]. Aziz et al. [19] used several oils—including epoxidized palm oil (EPO), and petroleum-based oils (PBOs) such as naphthenic oil (NO) and light mineral oil (LMO)—as additives for NR-based MREs. Although the limitation of the method is that the agglomeration of CIPs still occurs in the MRE samples, where it causes a decrease in crosslink density, PBO showed the potential to improve the dispersibility of magnetic particles and, thus, MR performance.

However, the elaborated investigation of the effects of PBOs at various ratios on the NR-based MREs can be considered limited, to the best of the authors’ knowledge. Previously, it has been reported that the addition of PBOs to NR compounds results in a small variation in curing behavior, including cure time and scorch time [10]. NO, LMO, PO, and AO are categorized as PBO products, and are effective for rubber processing. NO is an organic solvent and stable compound [29]. LMO and PO have been reported to enhance the processing properties, low-temperature properties, and dispersion of fillers, as well as reducing cost [10]. Meanwhile, AO with high aromatic contents can maintain the mechanical properties of MREs. A mixture of these PBOs may reduce cohesive forces between polymer chains due to their unsaturated rings and high reactivity with the rubber [10]. Subsequently, this may inhibit the energy from dissipating to the surroundings, leading to an increase in the MR effect as more energy can be stored [30]. PBO has also shown significant effects on the process of rubber compounding and the mechanical properties of the vulcanizate [31]. These oils are utilized to reduce mixing temperatures during processing, decrease the viscosity of rubber, prevent scorching or burning of the rubber compound, and modify the physical properties of the vulcanized rubber compound [31]. The addition of these oils is expected to enhance the dispersion ability of the magnetic particles, hence improving the MRE performance [1,16,32,33]. Moreover, it is expected that less energy will be dissipated as heat, thus enhancing the storage modulus and MR effect.

Therefore, this study aims to investigate and evaluate the influence of different ratios of PBOs on the performance of MREs with respect to physicochemical characteristics. The NR-based MREs were fabricated with different ratios of PBOs and prepared in isotropic conditions. The results of NR-based MREs featuring PBOs as dispersing aids—including cure characteristics, microstructure, thermal analysis, and magnetic properties—are discussed.

## 2. Materials and Methods

### 2.1. Materials Preparation

The natural rubber (NR) was used as a matrix for fabricating MRE samples. Carbonyl iron particles (CIPs) with irregular shapes were purchased from Sigma-Aldrich, Taufkirchen, Germany (AR grade). The density of the CIPs was 7.86 g/cm^3^, and 5–10 μm average particle size was used for the magnetic particles in this study. The petroleum-based oils (PBOs) used in this study included naphthenic oil (NO), light mineral oil (LMO), paraffin oil (PO), and aromatic oil (AO). Zinc oxide and n-cyclohexyl-2-benzothiazolesulfenamide (CBS) were prepared as the activator and accelerator, respectively.

### 2.2. Fabrication of MREs Samples

The formulation of the MRE compounds used in this study is presented in Table 1. Generally, there were three main stages of the mixing process in preparing all MRE samples using a conventional double-roll mill. The first stage was the addition of NR and additives, including zinc oxide, stearic acid, carbon black, NO, LMO, PO, and AO. At the second stage, sulfur and CBS were added until the NR compound was produced. The sulfur and accelerator were added at the end of the process to prevent premature vulcanization during compounding. Then, the NR compound and CIPs were fixed at 60 wt% and homogeneously mixed at the final stage. The final MRE compound with a thickness of 1.0 mm was fabricated via vulcanization. The vulcanization process was performed without a magnetic field at 150 °C for 30 min. The samples for the ratios of NO:AO, LMO:AO, and PO:AO were prepared accordingly at 100:0, 70:30, 50:50, and 30:70, as shown in Table 2. The MRE samples were named NO 1, NO 2, NO 3, NO 4, LMO 1, LMO 2, LMO 3, LMO 4, PO 1, PO 2, PO 3, and PO 4. The chemical structure of PBO can be found in Table 3. The oil molecule contains unsaturated rings, saturated rings, and paraffin side chains.

### 2.3. Cure Characteristics Analysis

The cure characteristics were determined using a moving die rheometer (MDR) (MDR 2000 on COM1) at a temperature of 150 °C for 30 min. A thin sheet of rubber with a thickness of 1.0 mm was heated by unidirectional conduction. The rubber sheet was placed between two dies, where the temperature was kept constant at 150 °C. The parameters of cure characteristics—including cure time (t_90_), scorch time (t_s1_), maximum torque (M_H_), minimum torque (M_L_), and torque difference (ΔM)—were determined. The vulcanization time of the MRE samples corresponded to the t_90_, which was calculated according to Equation (1). The percentage reversion (R_30_) at 30 min is defined in accordance with Equation (2) [34].

(1) $$\begin{array}{c} t_{90}=M_{L}+\frac{90}{100}\;(M_{H}\;-M_{L})\\ M_{L}:Minimum\;torque\;value\\ M_{H}:Maximum\;torque\;value \end{array}$$

(2) $$\begin{array}{c} R_{30}=\frac{M_{H}-M_{t}}{M_{H}}\times 100\%\\ M_{H}:\;\mathrm{Maximum}\;\mathrm{torque}\;\mathrm{value}\\ M_{t}:\mathrm{Torque}\;\mathrm{value}\;\mathrm{at}\;\mathrm{time}\;(30\;\min) \end{array}$$

### 2.4. Microstructure Observation

The microstructure of the MRE samples was observed using a field-emission scanning electron microscope (FESEM) (Model: Zeiss Supra55 VP) with a magnification of 500× and at an accelerating voltage of 5 kV throughout the experiment process. All samples were coated with gold to prevent charging during the observation process.

### 2.5. Magnetisation Test

The magnetic properties of MR materials were measured at room temperature under a magnetic field of −800 to 800 millitesla (mT) using a vibrating sample magnetometer (VSM) (Microsense FCM-10, USA). The samples were packed in the VSM sample holder at the range 30–40 mg and vibrated continuously during the analysis. The magnetic properties of the MRE samples were determined, including retentivity, coercivity, and saturation values.

### 2.6. Thermal Analysis

The thermal properties of the NR-based MREs were analyzed in a nitrogen atmosphere via thermogravimetric analysis (TGA). Thermal properties are those properties of a material that are related to its conductivity of heat. The samples were exposed to increased temperatures in the absence of a magnetic field. The mass of each MRE sample was measured in the range of 8–10 mg, based on standard sample preparation. The samples were heated from approximately 25 to 800 °C, under a heating rate of 10 °C/min.

## 3. Results and Discussion

### 3.1. Cure Characteristics

**Figure 1:**
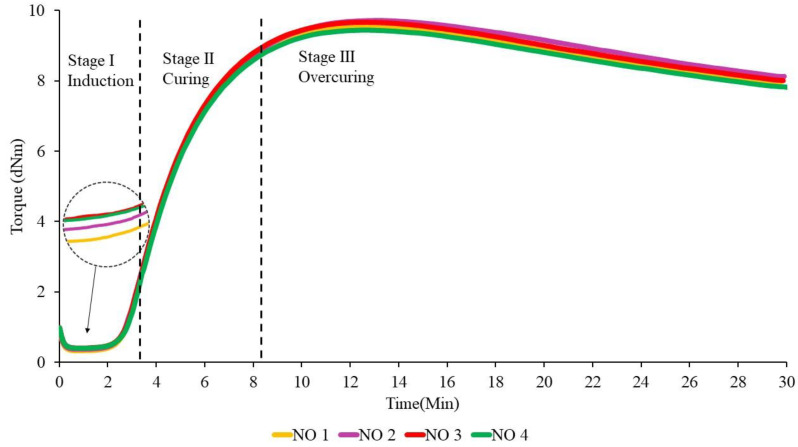
The curing curves of NR-based MREs for different ratios of NO:AO.

**Figure 2:**
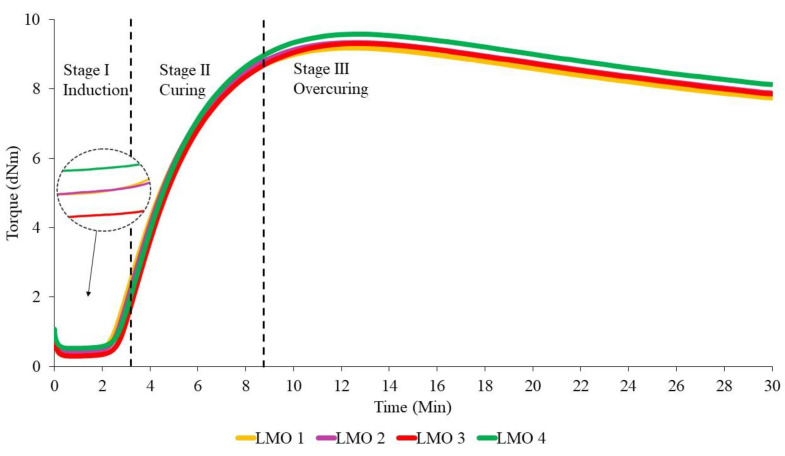
The curing curves of NR-based MREs for different ratios of LMO:AO.

**Figure 3:**
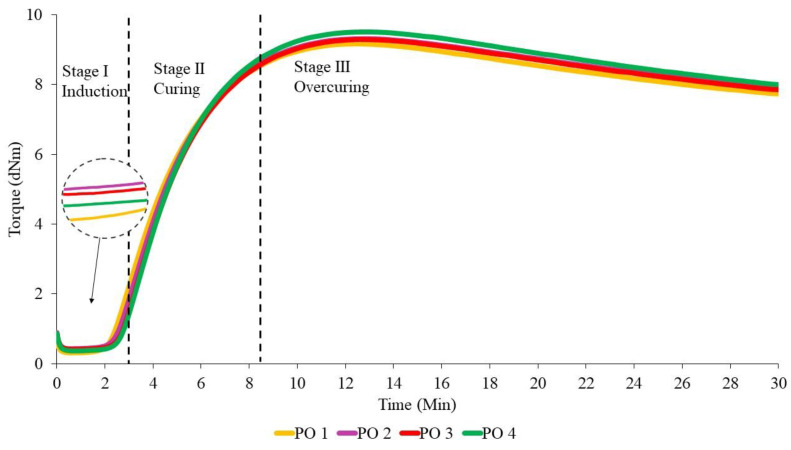
The curing curves of NR-based MREs for different ratios of PO:AO.

The effect of the addition of different ratios of PBOs is first discussed in this section, because the curing process is crucial for the MRE samples to become crosslinked. In general, the results show a slight change that can affect the thermal properties. Figure 1, Figure 2 and Figure 3 show the curing curves of NR-based MREs with three types of PBO: NO:AO, LMO:AO, and PO:AO, respectively. The experimental data were first filtered using the moving average method at every 30 data using Equation (3), where subscript n represents the variable at the n-th datum (n=1,…, N), x and $\bar{x}$ are the original data and the moving averaged data, respectively, and M is the size of the block. The moving standard deviation ($S_{d}$) is calculated based on the obtained moving average value as expressed in Equation (4) [35], where N is the total number of data. Generally, three stages could be obtained from the curing characterization curves: induction, curing, and overcuring. The first stage, which usually occurred within 0–3 min, is referred to as the induction stage, representing the slow chemical reaction between rubber and additives. The second stage is attributed to the curing period, wherein the torque increases from approximately 3–8.5 min due to the occurrence of rubber molecular chains forming network structures. The final stage, which occurred beyond 15 min, corresponds to overcuring. A decreasing trend of the graph obtained at this stage is known as a reversion curve. The reversion occurred due to the breakage of the crosslink bonds caused by the temperature, and this process depends on the rubber type, vulcanization agent, temperature, and content of polysulfide crosslinks in the rubber network [25,27,36]. Based on the literature, the reversion was measured using the calculation in Equation (2), whereby the lowest percentage of reversion is desirable for MRE materials [36,37,38,39].

(3) $${\bar{x}}_{n}=\frac{1}{M}\overset{M-1}{\underset{m=0}{\sum }}x_{\left(n+m\right)},\;m=0,\ldots ,M-1$$

(4) $$S_{d}=\sqrt{\;\frac{1}{N-1}\overset{N}{\underset{n=1}{\sum }}{\left({\bar{x}}_{n}-x_{n}\right)}^{2}},n=1,\ldots ,\;N$$

From Figure 1, we can see that the torque values slightly increased with the increase in the NO:AO ratio from 0:100 to 70:30 during the induction phase. Increasing the AO content provided no significant effect on the curing or reversion stages. However, to further evaluate the effects of PBO ratios, several crucial data were obtained from Figure 1, Figure 2 and Figure 3, and are presented in Table 4, Table 5 and Table 6. This is probably due to the breakdown of the chemical structure of oil between NR molecular chains. The addition of dispersion aids such as PBOs in the MRE samples weakens the intermolecular attraction forces between the polymer chains [26]. NO 2 indicated a low reversion process when NO was mixed with low amounts of AO, compared to NO 1, NO 3, and NO 4. This may have been a result of the softening characteristics of AO, which improved the workability of the rubber composition to provide good processability [4,20]. Apparently, the NR-based MREs showed good results in terms of reversion behavior when NO was mixed with low amounts of AO.

The curing curves for NR-based MREs containing different ratios of LMO are shown in Figure 2. The reversion process decreased when the ratio of LMO:AO increased. This could be due to the degradation of the polysulfide bonds in the rubber network when more AO was added to the sample. At 30 min, LMO 1 containing no AO showed the highest reversion. Meanwhile, the curves for other ratios—including LMO 2, LMO 3, and LMO 4—were overlapped, corresponding to insignificant effects on reversion. This may have been a result of the breakage of the crosslink bonds in the rubber chains during the mixing process. The results show that the crosslink bonds easily break without the presence of AO in the samples, and subsequently decrease the reversion curve. The sample with the presence of AO contributes to favorable reversion behavior, which corresponds to high resistance to the breakage of crosslink bonds.

Figure 3 depicts the curing curves for different ratios of PO:AO. Similar to NO:AO and LMO:AO ratios, all MRE samples using PO:AO exhibited reversion behavior after 15 min of the curing process. Increased reversion behavior was observed when the PO was mixed with high amounts of AO in the NR compounds. The reversion percentage of PO 2 was the lowest compared to PO 1, PO 3, and PO 4. This was due to the breakdown of paraffin side chains in the PO and AO at a ratio of 70:30, which resulted in decreased reversion in the NR network. In addition, AO has a good impact on the cure characteristics when it is mixed with PO. This may be due to the unsaturated ring compound in the oil, which is compatible with the NR network [11]. PO 4 showed increased reversion behavior when it was mixed with the AO in the MRE samples. Aromatic content in the MRE samples might interact with PO, resulting in an increased reversion curve.

The values of scorch time (t_s1_), cure time (t_90_), minimum torque (M_L_), maximum torque (M_H_), and torque value difference (ΔM) that were obtained from curing curves of different ratios of PBOs are summarized in Table 4, Table 5 and Table 6. The moving $S_{d}$ for all NR-based MRE samples with different ratios of oils in Table 4, Table 5 and Table 6 was found to be approximately <1 dNm for each sample. The value of $S_{d}$ was obtained from the torque value for each sample.

According to the data in Table 4, the addition of different ratios of PBOs affects the cure characteristics of the NR-based MREs. The value of scorch time (t_S1_) represents the period of time required for the safe processing of the rubber compound. Based on Table 4, NO 1, NO 2, and NO 3 showed very small effects on the scorch time, where the difference was only 0.01–0.02 min. This may have been due to the rising amount of additives during the induction stage to break down the intermolecular friction between rubber in the NR. Hence, the additives prevented the rubber chains from breaking down within the NR matrix, resulting in intermolecular friction and, consequently, causing the insignificant difference in the scorch time. Meanwhile, NO 4 samples indicated the highest scorch time of ~3.49 min due to the high AO content, which delayed the curing process. The long scorch time, corresponding to a slow chemical reaction between the rubber and additives, was favorable compared to the short scorch time in terms of providing enough processing safety. Meanwhile, the shortest scorch time had an influence on the crosslink, wherein the crosslinking started quickly in the compound [7,31,37].

The optimal cure time (t_90_) was equivalent to 8 min, which is the time required for the rubber compound to reach the state of curing 90% of the rubber, achieving optimal properties. Referring to Table 3, NO 2 demonstrated the highest cure time, with a value of 8.79 min. Meanwhile, NO 4 provided the lowest value, which was 8.54 min. It can clearly be seen that the presence of low amounts of AO can prolong the cure time. According to Neau et al. [40], three samples of NO were compared to AO, and all of the NO samples showed a longer cure time than AO due to the lower amount of sulfur compounds in the oil compared to AO. In addition, AO may accelerate the curing reaction in NR compounds, which contain a more reactive double bond. The presence of nitrogen and sulfur in the PBO is one of the factors that can accelerate the curing process and, consequently, affect the crosslink density [40,41].

The t_S1_ values showed that LMO 1 had the lowest scorch time (t_s1_) compared to other samples, at 3.32 min. This may be due to the poor compatibility of LMO with the rubber matrix during the mixing process. In this experiment, LMO 3—containing the same amounts of LMO and AO in phr—had the longest scorch time, while delaying the beginning of the vulcanization reactions [37]. However, LMO 4, which contained 3 phr of LMO and 7 phr of AO exhibited almost the same ts_1_ behavior as LMO 3. Unsaturated rings in the AO of the MRE samples might play important roles during mixing to delay the safety time.

Regarding the t_90_ values, different ratios of LMO in the MRE samples exhibited distinct vulcanization times. LMO 3 and LMO 4 had similar behavior, whereby the LMO mixed with AO in those samples presented an increase in vulcanization time. This might be explained by the reduced amounts of AO, which does not help much when it is mixed with LMO. However, in comparison, at t_90,_ LMO 4 exhibited the longest scorch time because of the high amounts of AO. These findings reflect the influence of different ratios of oil on the compatibility of rubber compounds. In addition, AO can accelerate the curing reaction in NR compounds, which contain more reactive double bonds [10,26].

The results showed that each different NR-based MRE sample exhibited different cure characteristics due to different ratios of PO:AO. The value of t_S1_ for PO 1 showed the lowest time, at 3.19 min, and contained 10 phr of PO in the sample. This may be due to the incompatibility of the PO with the NR compound during the curing process, leading to a decreased value of t_S1_. Meanwhile, PO 4 exhibited the highest t_S1_ as compared to PO 1, PO 2, and PO 3. The highest amount of AO mixed with the PO might delay the vulcanization reactions at the beginning of the process. However, PO 3 also exhibited similar behavior to PO 4. The high aromatic content in the sample caused an increasing scorch time, which resulted in good and safe processing.

It can be seen that the t_90_ of different ratios of PO increased when it was mixed with high amounts of AO. However, the PO 2 and PO 3 ratios did not significantly affect the vulcanization process, wherein both had similar values of vulcanization time. Referring to Table 6, PO 4 demonstrated the highest cure time, with a value of 8.59 min. Meanwhile, PO 1 provided the lowest cure time, at 8.28 min, while PO 2 had a cure time of 8.43 min. It can clearly be seen that the presence of a high amount of AO in the sample can prolong the cure time. The mixture of PO with AO based on the aromatic content of the oil might increase the cure time. It can be concluded that the addition of the AO to the other PBOs influenced the curing characteristics in terms of scorch time and optimal cure time.

### 3.2. Microstructure Observation

**Figure 4:**
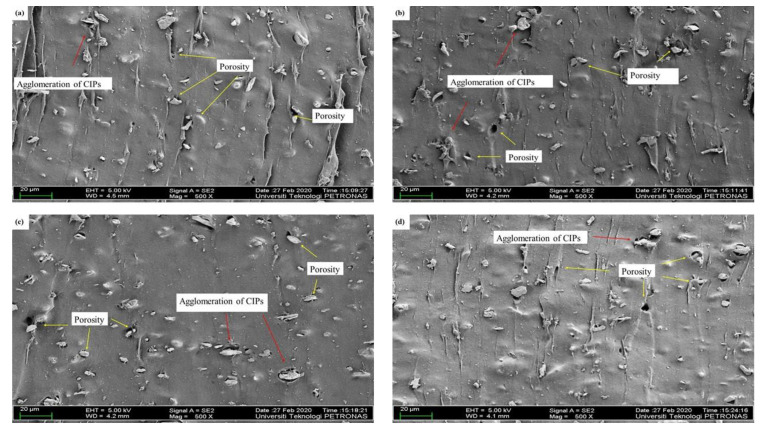
Microstructures of MREs for different ratios of NO:AO. (**a**) NO 1, (**b**) NO 2, (**c**) NO 3, and (**d**) NO 4.

Figure 4 illustrates the microstructure of isotropic MRE samples. All NR-based MRE samples exhibited a homogeneous distribution of CIPs. Clearly, the agglomeration of CIPs occurred in the MRE samples, as shown in Figure 4a–d. In general, it is known that during the early stages of the mixing process, NR presented high viscosity and elasticity. To acquire a homogeneous distribution of the CIPs, high shear forces were required during early mixing to disperse the CIPs and mitigate the agglomeration during the fabrication process. As shown in Figure 4a–d, increased porosity may occur due to the weak attraction between the NR, additives, and CIPs during the curing stage, consequently resulting in weak adhesion between the MRE sample components.

Based on Figure 4b–d, the MRE samples formed large agglomerations and many pores in the presence of more AO. This may have been due to the existence of high aromatic content in the AO. The high aromatic content in the AO had weak interactions with the NO, NR phase, and CIPs within the MREs, decreasing the crosslink density, as shown in Table 3. It can be concluded that the higher the aromatic content, the greater the possibility of agglomeration occurring. Meanwhile, Figure 4a shows that a large agglomeration was observed in the MRE sample containing NO without AO, and the porosity still occurred in the sample. However, the greater number of CIPs embedded within the NR phase indicated a better interface between the NR and CIPs. Clearly, CIPs could blend well with the matrix and the dispersing aids. This could be due to the high content of NO, which has good compatibility with NR and is more stable compared to AO. Consequently, the higher the ratio of NO to AO, the better the compatibility between the NR phase and the CIPs.

**Figure 5:**
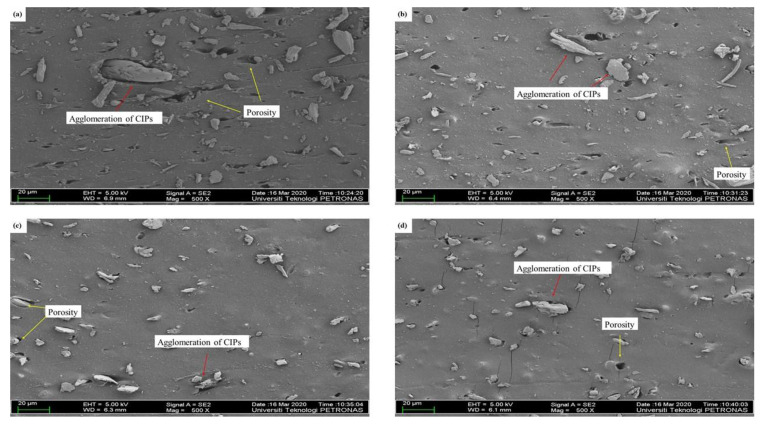
Microstructures of MREs for different ratios of LMO:AO. (**a**) LMO 1, (**b**) LMO 2, (**c**) LMO 3, and (**d**) LMO 4.

Referring to Figure 5a–d, the microstructures of different ratios of LMO:AO were randomly distributed within the MRE samples. Figure 5a illustrates the large agglomeration of CIPs in the absence of AO. This may be due to the incompatibility of the NR phase with the LMO, which resulted in a decrease in crosslink density in the absence of AO. LMO has a low density, which means it is less prone to interact with the NR phase and CIPs within the MREs. Meanwhile, Figure 5b–d show fewer agglomerations and decreased porosity in MRE samples containing the AO, as compared to Figure 5a. It can be seen that the AO helps to reduce agglomeration within MRE samples when it is mixed with the LMO. The CIPs can blend well when the LMO is mixed with the AO. The unsaturated ring content in the AO has a high reactivity, which enables it to easily interact with the NR and CIPs. It can be concluded that the best ratio of LMO:AO with respect to the MRE microstructure is found in the samples with high AO content, since less agglomeration and porosity were observed, leading to an increase in the crosslink density, as shown in Table 4.

**Figure 6:**
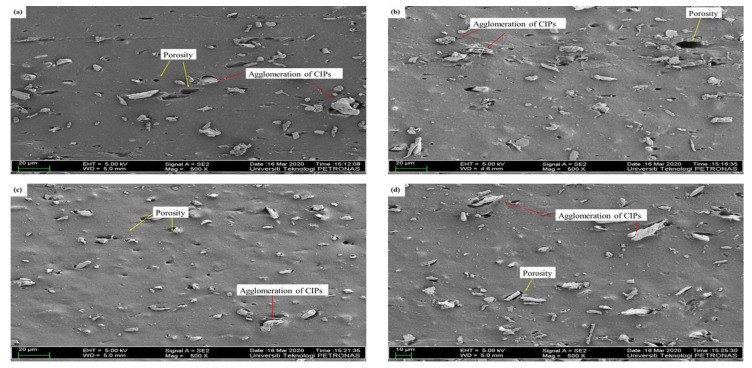
Microstructures of MREs for different ratios of PO:AO. (**a**) PO 1, (**b**) PO 2, (**c**) PO 3, and (**d**) PO 4.

The microstructure of the MREs’ cross-section for PO:AO was observed with different ratios, as shown in Figure 6a–d. All of the samples exhibited similar random CIP distribution within the MREs. It can be seen that the agglomerations and porosity still formed within the MREs. Figure 6a,b,d display large agglomeration within the MRE samples. Referring to Figure 6a,b, a large agglomeration is formed in the MREs due to the high levels of paraffin side chains. The paraffin side chains have no double bond, which leads to less reactivity within the MREs, and the incompatibility of the NR phase with the PO. Meanwhile, Figure 6c shows less agglomeration and porosity within the MREs when the ratio of PO:AO is 50:50, due to the existence of both aromatic and paraffinic content. At this ratio, the aromatic and paraffinic content contributed to well-blended CIPs in the NR phase. This ratio is considered when the PO and AO mixture is needed to reduce the agglomeration and porosity within the MREs. In conclusion, PO contributed to a reduction in agglomeration within MRE samples.

### 3.3. Magnetic Properties

Magnetic properties in MREs are important for evaluating the bonding between the particles and the matrix in MREs that produce a high MR effect and good mechanical properties [15]. The magnetization curves exhibited narrow magnetic hysteresis loops for all MRE samples, corresponding to soft magnetic characteristics. The trend of the graph is similar for all samples, where the magnetization curves increase as the magnetic field strength increases from −8000 mT to 8000 mT. Then, the slope begins to decrease when the CIPs in the MRE samples reach their saturated condition, where the M_S_ value is obtained. The parameters that influenced the magnetic properties of the MRE samples were saturation magnetization (M_S_), coercivity (H_C_), and retentivity magnetization (M_R_), the values of which are depicted in Table 7, Table 8 and Table 9. As seen from the magnetization curves, the values of M_R_ and H_C_ in MRE samples can be obtained at the vertical and horizontal axes, respectively. Coercivity is defined as the magnetic field that is required to drive the reverse magnetization after being saturated. Meanwhile, the retentivity is a measure of magnetization that remains after the applied magnetic field is released.

**Figure 7:**
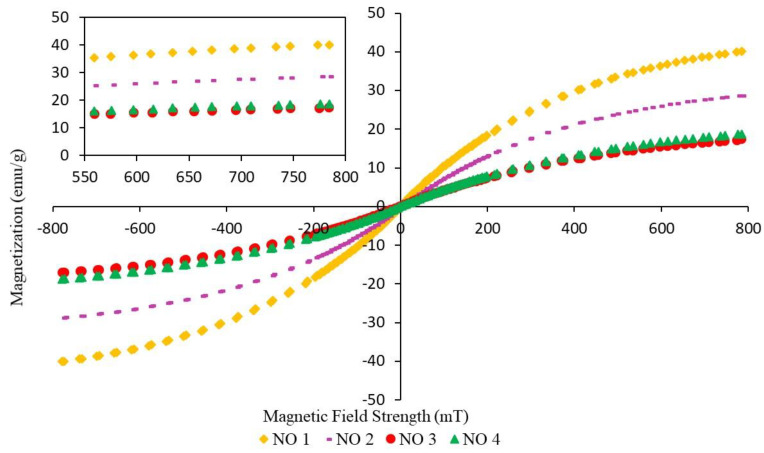
Magnetization curves of NR-based MREs for different ratios of NO:AO.

Figure 7 shows the magnetization curves of NR-based MREs with different ratios of NO:AO. The magnetization curves of NO 3, where the ratio of PBO was 50:50, showed the lowest magnetization. Meanwhile, NO 1 had the highest magnetization curves compared to NO 2, NO 3 and NO 4, due to the high content of NO in the sample, which showed better magnetic properties.

**Figure 8:**
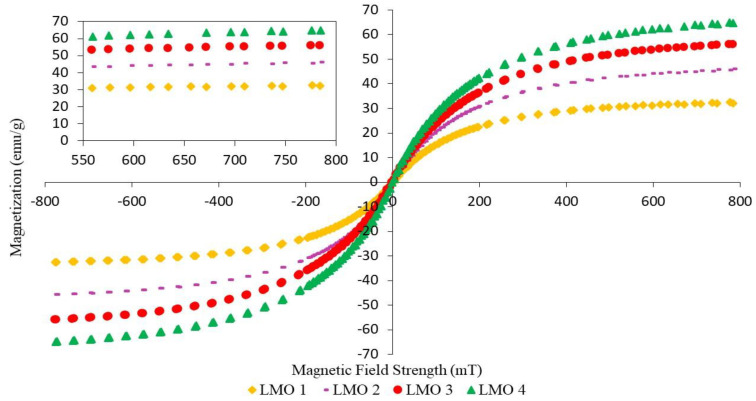
Magnetization curves of NR-based MREs for different ratios of LMO:AO.

Figure 8 shows the magnetic hysteresis loops for MRE samples with different ratios of LMO:AO. It can be observed that LMO 4 exhibited the highest value of magnetic saturation as compared to LMO 1, LMO 2, and LMO 3. This was probably due to the high content of AO mixed with the LMO. The content of AO might help LMO in terms of interaction and compatibility between CIPs and the NR matrix, resulting in better dispersion of CIPs within the MREs [42].

**Figure 9:**
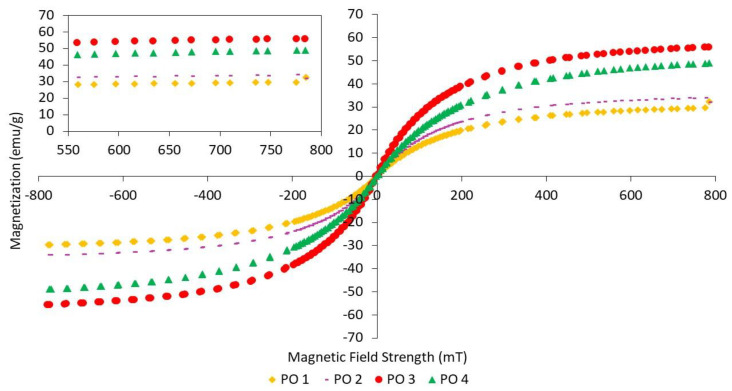
Magnetization curves of NR-based MREs for different ratios of PO:AO.

The magnetization curves for MRE samples with different ratios of PO:AO are shown in Figure 9. PO 1 shows the lowest magnetic saturation in the absence of the AO content. Meanwhile, PO 3 represents the highest value of magnetic saturation as compared to PO 1, PO 2, and PO 4. PO 3 might be the best ratio for magnetic properties due to the interaction of PO and AO when mixed one another at a ratio of 50:50.

Table 7 represents the M_S_, M_R_, and H_C_ for different ratios of PBO, where the values are obtained from Figure 7. Apparently, NO 1 showed good magnetic properties, whereby the sample produced the highest magnetic saturation of 40.06 emu/g. The addition of AO at ratios of 70:30 and 50:50 resulted in a decrease in the value of magnetic saturation from 28.73 to 17.23 emu/g. However, magnetic saturation was slightly increased in the case of the NR-based MRE sample with a ratio of 70:30. It can be seen that, when not combining NO with AO, NR-based MRE samples exhibited better magnetic properties as compared to the samples containing AO. This could be due to the compatibility of the oil with the NR matrix, which offers a better dispersion of CIPs compared to NO 3. The compatibility and interaction between the matrix and the filler are the predominant factors in improving the performance of MREs. The presence of NO contributed to the enhancement of the magnetic properties of MREs, due to the compatibility between oil and NR. This is because NO is a stable compound due to its high levels of saturated rings, resulting in the CIPs becoming homogeneously dispersed in the morphological observation [29].

Table 8 shows the magnetic properties of MREs for different ratios of LMO:AO. The addition of AO to LMO contributes to an improvement in magnetic properties, increasing the magnetic saturation from 45.90 to 64.87 emu/g. This improvement of magnetic properties could be due to the good compatibility between LMO and AO, which contain similar ring structures. Good compatibility helped in producing good distribution of CIPs, and subsequently contributed to high magnetic interaction among CIPs. Apparently, using LMO alone as a dispersing aid produced the lowest value of magnetic saturation, at ~32.53 emu/g. This was probably due to the poor dispersion of CIPs within NR-based MREs, as depicted in Figure 5a. When LMO is mixed with a high content of AO, the unsaturated ring in AO may cooperate with the low density of LMO. The similar unsaturated rings of AO and LMO have a good interaction between NR and CIPs; as a result, incorporating a large amount of AO into LMO may improve compatibility within MREs, resulting in better CIP dispersion. This is confirmed by the microstructure shown in Figure 5d, which represents a better dispersion of CIPs.

As stated in Table 9, the MREs with different ratios of PO:AO showed different effects on magnetic properties. The decreasing content of PO from 100 to 50 wt% resulted in an increase in magnetic saturation from 31.00 to 55.71 emu/g. Meanwhile, the 30% content of PO at a ratio of 30:70 showed a decrease from 55.71 to 48.94 emu/g. The presence of unsaturated rings associated with double bonds in AO is compatible with the paraffinic side chains that exist in PO. Compatibility between PO and AO reached its maximum when the ratio was 50:50, as the magnetic saturation started to decrease at a ratio of 30:70. PO 3 showed the best results in the enhancement of magnetic properties, due to the CIPs being well dispersed. The highest magnetic properties among the different ratios of PBO above were those of LMO 4. This can be seen in the microstructure observation in Figure 5d, where the CIPs were well dispersed within the NR matrix. Therefore, it can be said that using PBO as a dispersing aid might help in producing a better dispersion of CIPs, depending on the ratios.

### 3.4. Thermal Characteristics

**Figure 10:**
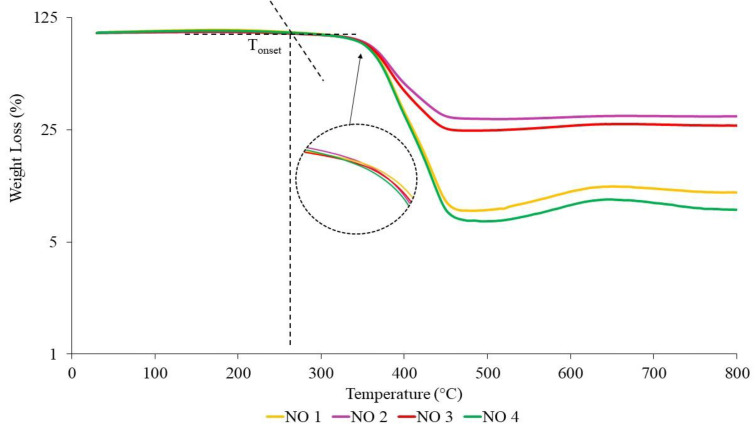
TGA curves of NR-based MREs for different ratios of NO:AO.

**Figure 11:**
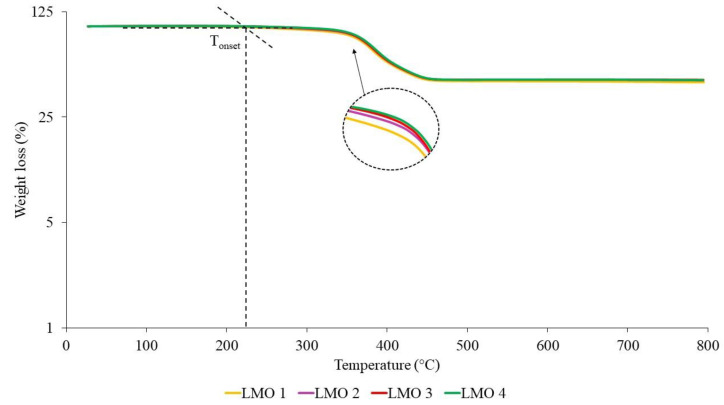
TGA curves of NR-based MREs for different ratios of LMO:AO.

**Figure 12:**
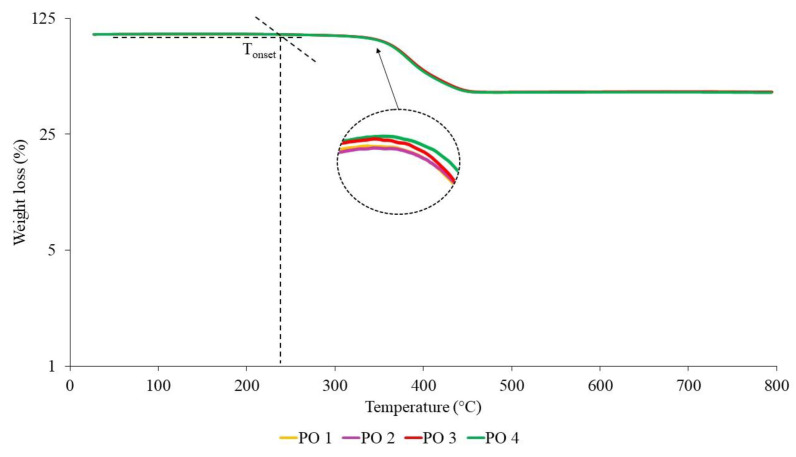
TGA curves of NR-based MREs for different ratios of PO:AO.

**Figure 13:**
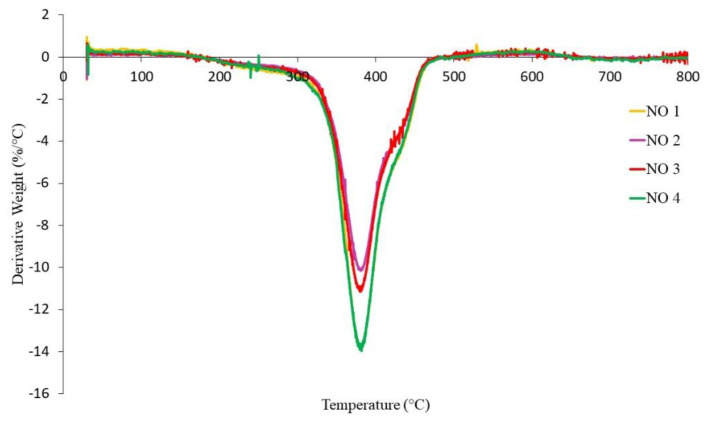
DTG curves of NR-based MREs for different ratios of NO:AO.

**Figure 14:**
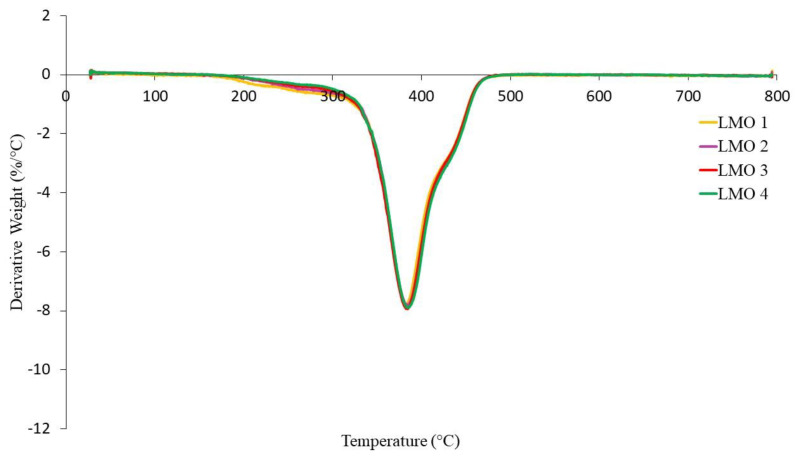
DTG curves of NR-based MREs for different ratios of LMO:AO.

**Figure 15:**
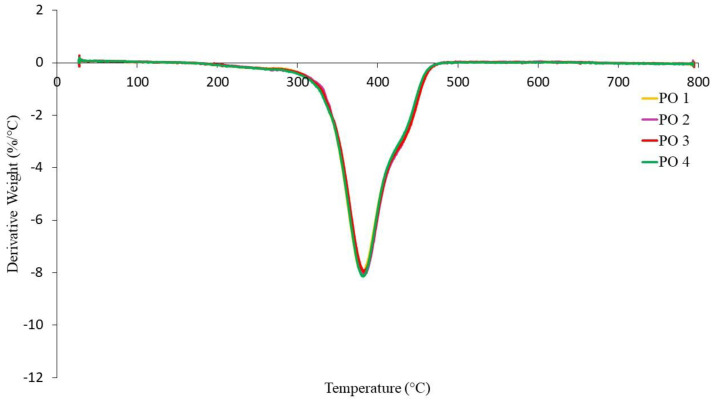
DTG curves of NR-based MREs for different ratios of PO:AO.

The TGA curves of NR-based MREs with different ratios of PBOs measured in a nitrogen atmosphere are demonstrated in Figure 10, Figure 11 and Figure 12. Meanwhile, differential thermal gravimetric (DTG) curves are shown in Figure 13, Figure 14 and Figure 15. Two decompositions occur from the TGA curves. At stage I of all samples, it was found that the TGA curves decreased slightly, and no peaks were observed in the DTG curves, as shown in Figure 13, Figure 14 and Figure 15. Stage I was defined as the degradation of volatile matter with certain additives that are added to the samples during the compounding process. Meanwhile, the degradation of polymers for NR in this temperature range is referred to as stage II; during this stage, the TGA curves were dramatically decreased, and the peaks were observed as illustrated in Figure 10, Figure 11 and Figure 12. In general, the weight loss of NR-based MREs corresponded to the degradation of volatile matter and the NR matrix.

Based on Figure 10, the temperature ranges for stage I and stage II of NR-based MRE samples using NO:AO are 260 to 343 °C and 343 to 465 °C, respectively. Stage II was associated with the endothermic peaks observed in the DTG curves at all PBO ratios.

Based on Figure 10, NO 2 showed the lowest total weight loss of approximately 70 wt% due to the increasing of crosslink density within MRE samples, which could have improved the thermal resistance of the rubber compositions. The presence of a low amount of AO mixed with the NO caused an increase in crosslink density to occur during the curing process. Thus, the crosslink density increased, and the rubber chains were delayed, resulting in decrease in weight loss. In addition, this outcome was in parallel with the results of curing characterization, where NO 2 showed a low percentage of reversion during the curing process. This sample corresponded to the low amount of AO associated with the narrow peak that occurred in Figure 10. The peaks occurred due to the heat absorbed and consequent breaking of the crosslink rubber chains. The presence of a high crosslink density caused the low weight loss. As such, NO 2 showed a better result in terms of thermal decomposition and a broader peak when NO was mixed with low amounts of AO as compared to other samples. NO 3 exhibits low weight loss at 75 wt% when mixed with the same amount of AO at a 50:50 ratio; this could be due to the increase in its crosslink density. This could be explained by the interaction between rubber and additives within MRE samples at a 50:50 ratio. The increase in the crosslink density resulted in decreased weight loss.

Meanwhile, NO 1 and NO 4 showed similar behavior, with high weight loss of approximately 80–90 wt% as compared to the other samples. This may have been a result of the decrease in crosslink density occurring in MRE samples during the curing process, resulting in reduced thermal stability. Hence, the amount of oil at the ratios of NO 1 and NO 4 might cause the degradation reactions to occur, as a result of which the crosslink bonds easily break. The material is easily decomposed due to the decrease in the formation of crosslink bonds in the rubber compound, making it break more easily. However, the effects of different ratios of PBO on the thermal degradation of MREs are different due to the presence of saturated rings in the NO [39,40,43,44]. Since the mass of CIPs was constant (60 wt%), and CIPs can only decompose at temperatures beyond 1000 °C, it can be deduced that the weight loss of NR-based MREs was predominantly influenced by different ratios of PBO. Figure 13 shows the endothermic peaks in the DTG curves of NR-based MREs for different ratios of NO:AO. The peaks occurred at stage II from 343 to 465 °C due to the polymer degradation of the NR matrix. The broad peaks that were obtained in the DTG graph was showed similar trends. This could be due to the presence of unsaturated rings and saturated rings and consequently, the heat absorbed when the rubber chains were breaking. 

Figure 11 shows TGA curves for different ratios of LMO:AO. The degradation in TGA curves can be observed at a temperature range of 200–500 °C. Stages I and II occurred at temperature ranges of 200 to 324 °C and 324 to 475 °C, respectively. All samples for different ratios of LMO:AO showed insignificant effects on the TGA curves, where the weight loss was shown to be approximately 60–65 wt%. This may have been due to the similar rings present in the AO and LMO, which are known as unsaturated rings. These rings showed compatibility when LMO was mixed with AO, which resulted in an insignificant effect on the degradation of the MREs. Figure 14 shows the endothermic peaks in the DTG curves. The peaks occurred from 324 to 475 °C due to the polymer degradation of the NR matrix. The broad peaks that were obtained in the DTG graph for different ratios of LMO:AO showed similar trends. This could be due to the heat absorbed when the rubber chains were breaking. However, all samples exhibited similar trends.

Figure 12 shows the TGA curves for the different ratios of PO:AO. The significant weight loss for these samples occurred when the temperature range was at 200–500 °C. Referring to the TGA curves, stage I and stage II started at temperatures of 220 to 327 °C and 327 to 472 °C, respectively. The results of PO:AO samples showed an insignificant effect and similar behavior with respect to temperature. These results were identical to those of the LMO:AO samples, resulting in an insignificant effect. The addition of AO to PO exhibits good compatibility, as AO possesses an unsaturated ring and PO has paraffin side chains. The chemical structures of PO and AO caused high reactivity within MRE samples. Figure 15 shows DTG curves for different ratios of PO:AO, and endothermic peaks were obtained at stage II. The DTG peaks were observed due to the breaking of crosslinked rubber chains. Then, the broad peaks showed similar trends in the DTG peaks for all samples. This could be due to the interaction of paraffin side chains with unsaturated rings, which caused the breaking of the crosslinks of rubber chains and, consequently, showed similar behavior in terms of weight loss.

The degradation temperature of MREs with different ratios of PBO is demonstrated in Table 10, Table 11 and Table 12. T_onset_ represented the temperature when the NR matrix started to degrade, while T_end_ corresponds to the end of the degradation. T_max1_ is the maximal temperature for the thermal degradation rate of PBO. Meanwhile, T_max2_ is the maximal temperature for the thermal degradation rate of NR. Apparently, NO 1 showed the lowest value of T_onset_ at 260 °C without mixing with AO, increasing to 300 °C when NO was mixed with a low amount of AO at a ratio of 70:30 (NO 2). The addition of AO at ratios of 50:50 and 70:30 resulted in a decreasing value of T_onset_ from 290 to 280 °C. However, the T_onset_ was slightly increased in the case of NR-based MRE samples with ratios of 50:50. It could be said that mixing NO with AO at a ratio of 50:50 for NR-based MRE samples exhibited a better thermal degradation compared to the samples containing high amounts of AO. Hence, the high content of NO mixed with low amounts of AO delayed the occurrence of thermal degradation. The NO mixed with low amounts of AO showed the potential to improve the thermal properties, contradicting the results of the magnetic properties.

Table 11 shows the thermal degradation temperatures for different ratios of LMO:AO. The addition of AO to LMO with increasing ratios—from 0:100 (LMO 1) to 70:30 (LMO 4)—contributed to a better resistance to thermal degradation, where LMO 4 showed the highest value of T_onset_, followed by LMO 3, LMO 2, and LMO 1, with values of 258, 242, 226 and 223 °C, respectively. The T_onset_ for all samples of LMO:AO increased with the increasing content of AO. The effects of different ratios of LMO:AO on the thermal degradation of MREs could be due to the presence of unsaturated rings, as explained in the discussion of TGA curves above. The heat transfer is expected to be faster with low amounts of AO, so the material will decompose at lower temperatures.

Thermal degradation for different ratios of PO:AO within MRE samples is shown in Table 12. The sample of PO without AO showed the lowest value of T_onset_, at 227 °C, where thermal degradation decreased dramatically. Meanwhile, the samples of PO 2, PO 3, and PO 4, where PO was mixed with increasing amounts of AO, showed an increasing thermal degradation temperature, where the values of T_onset_ were 235, 242, and 260 °C, respectively. These results contributed to delayed thermal degradation. It can be seen that AO delayed the degradation of the sample due to the unsaturated ring in AO, which contains a more reactive double bond within the NR matrix. Apparently, the best ratio of this sample to obtain thermal properties is PO 4, as compared to PO 1, PO 2, and PO 3. It should be noted that the thermal degradation increased with increasing amounts of AO content. Overall, NO 2 showed better thermal properties, since the value of T_onset_ was delayed at 300 °C as compared to other samples. Furthermore, this suggests that different ratios of PBO particularly NO to AO have a great influence on the thermal properties and weight loss of NR.

## 4. Conclusions

In this research, the influence of different ratios of PBOs as dispersing aids with 60 wt% CIPs on the physicochemical properties of MREs was investigated. The curing characteristics, microstructure, and magnetic and thermal properties of MREs with different ratios of PBOs were analyzed. The microstructural results revealed the formation of CIPs in an isotropic state. With the addition of the PBOs as dispersing aids, the gap between CIPs and the agglomeration of magnetic particles caused a reduction in the cohesive force of crosslinked monomers within the MREs. The PBO ratios with high content of AO—particularly for LMO—contributed to good magnetic properties, with the highest Ms value of 64.87 emu/g, due to the well-dispersed CIPs within the NR matrix, which are favorable for MRE applications since MREs are exposed to various magnetic field strengths. In terms of thermal properties, NO 2—which was mixed with a low amount of AO—contributed to better thermal properties, with the lowest weight loss of 70 wt% and the highest T_onset_ of 300 °C. Hence, this sample is desirable for MRE applications that are related to temperature. Apparently, LMO 4 was the best ratio in terms of curing characteristics and magnetic properties. In a nutshell, the PBOs had good compatibility and greatly influenced the physicochemical characteristics of NR-based MREs when they were mixed with other PBOs. In particular, the results presented in this work contribute useful knowledge, in that PBO ratios showed different effects depending on the analyzed characteristics. It should finally be remarked that the different ratios of PBO can alter the physicochemical properties of the NR-based MREs, which are considered a crucial part of MRE performance. The findings gained from this study could constitute useful guidance in fabricating NR-based MREs. In addition, comprehensive experimental studies on topics such as magnetic-field-independent rheological properties, including the MR effects of MREs, will be very useful to further evaluate MR performance using PBOs.

## Figures and Tables

**Table 1:** Formulation of compound ingredients.

Compound Ingredients	Function	Amount (phr)
Natural rubber	Matrix	100
Carbon black	Filler	19
Zinc oxide	Activator	5
Stearic acid	Activator	2
Sulfur	Crosslinking agent	2.3
CBS	Accelerator	0.8

**Table 2:** Compound ingredients for different ratios of PBO.

**Sample**	**Ratio of PBO**	**NO (phr)**	**AO (phr)**
NO 1	100:0	10	0
NO 2	70:30	7	3
NO 3	50:50	5	5
NO 4	30:70	3	7
**Sample**	**Ratio of PBO**	**LMO (phr)**	**AO (phr)**
LMO 1	100:0	10	0
LMO 2	70:30	7	3
LMO 3	50:50	5	5
LMO 4	30:70	3	7
**Sample**	**Ratio of PBO**	**PO (phr)**	**AO (phr)**
PO 1	100:0	10	0
PO 2	70:30	7	3
PO 3	50:50	5	5
PO 4	30:70	3	7

phr = parts per hundred of rubber.

**Table 3:** The chemical structure of PBO.

Chemical Structure		
(a) Unsaturated rings (Aromatic oil and light mineral oil)	(b) Paraffin side chains (Paraffin oil)	(c) Saturated rings (Naphthenic oil)
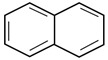	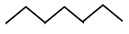	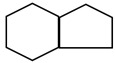

**Table 4:** Cure characteristics for different ratios of NO:AO.

Sample	t_S1_ (min)	t_90_ (min)	M_L_ (dNm)	M_H_ (dNm)	ΔM	R_30_ (%)	Standard Deviation (dNm)
NO 1	3.42	8.64	0.32	9.57	9.25	16.72	0.09
NO 2	3.41	8.79	0.36	9.73	9.37	16.44	0.09
NO 3	3.43	8.75	0.40	9.68	9.28	17.25	0.09
NO 4	3.49	8.54	0.39	9.45	9.06	17.24	0.09

**Table 5:** Cure characteristics for different ratios of LMO:AO.

Sample	t_S1_ (min)	t_90_ (min)	M_L_ (dNm)	M_H_ (dNm)	ΔM	R_30_ (%)	Standard Deviation (dNm)
LMO 1	3.32	8.31	0.40	9.19	8.79	15.78	0.12
LMO 2	3.41	8.44	0.40	9.34	8.94	15.68	0.12
LMO 3	3.58	8.41	0.29	9.32	9.03	15.67	0.11
LMO 4	3.55	8.68	0.51	9.59	9.08	15.22	0.13

**Table 6:** Cure characteristics for different ratios of PO:AO.

Sample	t_S1_ (min)	t_90_ (min)	M_L_ (dNm)	M_H_ (dNm)	ΔM	R_30_ (%)	Standard Deviation (dNm)
PO 1	3.19	8.28	0.30	9.17	8.87	15.59	0.13
PO 2	3.40	8.43	0.42	9.32	8.90	15.13	0.11
PO 3	3.54	8.41	0.40	9.30	8.90	15.48	0.11
PO 4	3.55	8.59	0.36	9.51	9.15	15.88	0.11

**Table 7:** Magnetic properties of MREs for different ratios of NO:AO.

Samples	Ms (emu/g)	M_R_ (emu/g)	Hc (G)
NO 1	40.06	0.18	16.38
NO 2	28.73	0.13	15.57
NO 3	17.23	0.075	16.77
NO 4	18.74	0.078	16.36

**Table 8:** Magnetic properties of MREs for different ratios of LMO:AO.

Samples	Ms (emu/g)	M_R_ (emu/g)	Hc (G)
LMO 1	32.53	0.30	14.17
LMO 2	45.90	0.42	14.40
LMO 3	55.98	0.42	13.78
LMO 4	64.87	0.52	13.48

**Table 9:** Magnetic properties of MREs for different ratios of PO:AO.

Samples	Ms (emu/g)	M_R_ (emu/g)	Hc (G)
PO 1	31.00	0.26	14.55
PO 2	34.06	0.33	14.59
PO 3	55.71	0.52	14.35
PO 4	48.94	0.39	15.06

**Table 10:** Thermal degradation temperature of MREs with different ratios of NO:AO.

Samples	T_onset_ (°C)	T_max1_ (°C)	T_max2_ (°C)	T_end_ (°C)
NO 1	260	318	377	450
NO 2	300	343	382	465
NO 3	290	331	379	457
NO 4	280	328	378	454

**Table 11:** Thermal degradation temperature of MREs with different ratios of LMO:AO.

Samples	T_onset_ (°C)	T_max1_ (°C)	T_max2_ (°C)	T_end_ (°C)
LMO 1	223	319	384	468
LMO 2	226	322	388	471
LMO 3	242	321	387	474
LMO 4	258	324	389	475

**Table 12:** Thermal degradation temperature of MREs with different ratios of PO:AO.

Samples	T_onset_ (°C)	T_max1_ (°C)	T_max2_ (°C)	T_end_ (°C)
PO 1	227	363	424	466
PO 2	235	366	427	472
PO 3	242	368	437	475
PO 4	260	370	440	489

## Data Availability

Not applicable.
